# Internal Herniation of Small Bowel Underneath Iliac Vascular Axis After Pelvic Surgery: A Systematic Review

**DOI:** 10.7759/cureus.42960

**Published:** 2023-08-04

**Authors:** Aizaz Khalid, Anza Ashraf, Mohamed A Salman, Richard C Newton

**Affiliations:** 1 General Surgery, University Hospitals Sussex National Health Service (NHS) Foundation Trust, Chichester, GBR; 2 General Surgery/Urology, Frimley Health National Health Service (NHS) Foundation Trust, Slough, GBR; 3 General Surgery, KasrAlainy School of Medicine, Cairo University, Cairo, EGY

**Keywords:** surgical repair of hernia, post-op complications, pelvic lymph node dissection, iliac artery, strangulated internal hernia

## Abstract

Internal abdominal hernias are rare entities that most commonly present with acute small bowel obstruction. These hernias can be congenital or acquired. While congenital hernias are considered the most common type, acquired hernias are becoming more common. Recently, a rare type of internal herniation has been reported underneath iliac vasculature in patients who have undergone pelvic lymph node dissection in the past. This study was carried out to assess the prevalence of this rare type of internal hernia.

Two reviewers searched the literature in three online databases using the Cochrane methodology for systematic reviews. The search of databases yielded 70 articles. The studies which reported internal herniation underneath iliac vasculature were included. Studies that reported herniation underneath other pelvic organs or vasculature were excluded. After screening, 17 articles were deemed suitable and selected.

All 17 cases reviewed underwent pelvic lymph node dissection in the past. The median latency period between index surgery and clinical presentation with the incarcerated hernia was 20 months. All 17 cases were managed surgically with small bowel resection carried out in 13 cases. Eleven authors reported closing the hernia defect with various techniques, while five decided not the close it. All 17 cases were alive at the time of discharge from the hospital, with a mean hospital stay of 12.7 days.

Given our findings, there should be a high index of suspicion of internal hernia in patients presenting with small bowel obstruction with a history of pelvic lymph node dissection. In our review, internal herniation was always preceded by pelvic lymph node dissection, so the closure of the peritoneum should be considered while pelvic lymph node dissection is carried out.

## Introduction and background

An internal abdominal herniation is the protrusion of viscera through an opening in the peritoneum or mesentery [1]. The true incidence of internal hernia is not known, but it does occur more commonly than it is generally believed. The autopsy incidence of internal hernias is between 0.2% and 0.9% [2]. While small hernias can remain asymptomatic, they can become incarcerated leading to strangulation and ischemia. This is the most common mode of presentation for an internal hernia, constituting up to 4.1% of all cases presenting with acute small bowel obstruction [3].

Because of its rare nature, the diagnosis of internal hernia is a challenge for both clinicians and radiologists [4]. The classification of internal hernias devised by Ghahremani [2] in 1994, is well accepted. According to this classification, internal hernias are divided into six main groups: paraduodenal hernias (most common), hernias through the foramen of Winslow, transmesenteric hernias, pericecal hernias, intersigmoid hernias, and perivesical hernias. While classically congenital hernias were considered to be the predominant type, acquired hernias are becoming more prevalent [5].

The authors’ personal experience with internal hernia is reported in Khalid et al. [6]. We found a case of an internal hernia underneath the external iliac artery. This sort of hernia is very rare and does not fit into any of the classification categories. Therefore, a systematic review was conducted to explore the prevalence of internal herniation of the small bowel underneath the iliac vasculature.

## Review

Methodology

**Figure 1:**
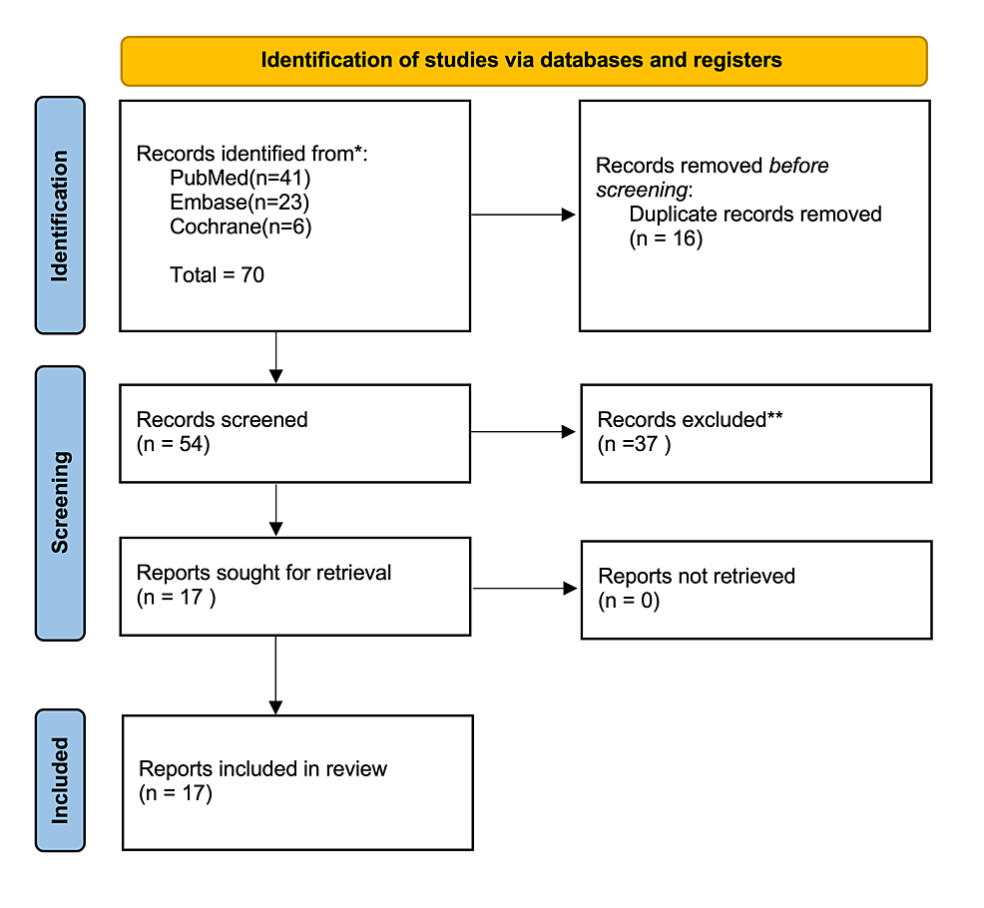
Study methodology used for the systemic Review (PRISMA). PRISMA, Preferred Reporting Items for Systemic Review and Meta-Analyses

A literature search of three online databases, namely, PubMed (1978 to present), Embase (1974 to present), and Cochrane CENTRAL (1988 to present) was conducted using the keywords ‘internal hernia’ which was cross-matched with ‘iliac artery.’ This search was conducted on July 13th, 2023. Duplicates were removed, yielding 54 records. The inclusion criteria selected were any articles that reported internal herniation of the bowel underneath the common iliac, external iliac, or internal iliac vessels. Studies with internal herniation underneath other pelvic organs or vasculature were excluded. All full texts were retrieved and 17 articles were found to be included in this review. Search screening and article shortlisting were performed using the Preferred Reporting Items for Systemic Reviews and Meta-Analysis (PRISMA) methodology (Figure 1) [7].

The 17 articles selected for our study were reviewed by two members of the team (AK, AA). Data from the selected articles, including index surgery, latency from previous surgery to complication, and method of closure, are tabulated for a detailed review (Table 1).

**Table 1:** Cases reported with internal herniation of small bowel underneath iliac vasculature. Latency refers to the time period between the previous surgery and presentation to the hospital. Hlos, hospital length of stay; TAH, total abdominal hysterectomy; ePLND, extended pelvic lymph node dissection

Study	Patient, Initial pathology	Previous surgery	Latency	Operative approach	Operative findings	Vascular relation	Closure of defect	Hlos (days)
Guba et al. 1978 [8]	52-year-old male, testicular teratoma	Radical retroperitoneal lymphadenectomy	4 months	Open procedure, small bowel resection with ileostomy	90% small bowel herniated with distal ileal perforation	Right common iliac artery	Free peritoneal graft	66
Kim et al. 2008 [9]	67-year-old female, cervical cancer	Laparoscopic radical hysterectomy, pelvic lymphadenectomy and bilateral salpingo-oopherectomy.	3 months	Open procedure, small bowel resection with anastomosis	Gangrenous ileum herniating between iliac vessels	Right external iliac	Free peritoneal graft	9
Dumont et al. 2013 [10]	56-year-old female, ovarian carcinoma	Laparotomy TAH, appendicectomy, omentectomy and retroperitoneal lymphadenectomy.	4 years	Laparoscopic reduction of internal hernia	Strangulated small bowel which was viable after reduction	Left external iliac artery	Not closed	2
Ardelt et al. 2013 [11]	39-year-old female, cervical carcinoma	Laparoscopic trachelectomy with pelvic lymphadenectomy.	2 years	Laparoscopic to open procedure, small bowel resection, and anastomosis	50 cm small bowel herniation with ischemic jejunum	Right common iliac artery	Collagen patch	6
Pridjian et al. 2015 [12]	50-year-old male, bladder carcinoma	Robot assisted partial cystectomy and ePLND	5 months	Open procedure, small bowel resection with anastomosis	Closed loops small bowel obstruction with necrotic ileum	Right common iliac artery	Peritoneal flaps	Not reported
Viktorin-Baier et al. 2016 [13]	50-year-old male, prostate carcinoma	Robot assisted prostatectomy with ePLND	12 months	Open procedure, small bowel resection with anastomosis, resection anastomosis of the artery	1.6 m small bowel incarcerated	Left external iliac artery with thrombus inside the artery	Collagen patch	13
Kambiz et al. 2018 [14]	64-year-old male, prostate carcinoma	Robot assisted prostatectomy with ePLND	12 months	Laparoscopic to open procedure, small bowel resection, and anastomosis	Necrotic small bowel herniated	Right external iliac artery	Not reported	Not reported
Ninomiya et al. 2019 [15]	72-year-old male, prostate carcinoma	Robot assisted prostatectomy with ePLND	2 months	Open procedure, small bowel resection with anastomosis	120 cm necrotic ileum herniated	Left external iliac artery	Not closed	10
Frostberg et al. 2019 [16]	65-year-old male, prostate carcinoma	Robot assisted radical Prostatectomy with ePLND	15 months	Open procedure, small bowel resection with ileostomy	70 cm ischemic small bowel herniated	Left external iliac artery	Not closed	Not reported
Felix et al. 2020 [17]	68-year-old female, endometrial carcinoma	Laparoscopic hysterectomy, pelvic lymphadenectomy and bilateral salpingo-oopherectomy.	7 years	Open procedure, small bowel resection with anastomosis	50 cm incarcerated ileum herniated	Right external iliac artery	Suture closure	5
Hishikawa et al. 2021 [18]	67-year-old female, ovarian cancer	Hysterectomy, bilateral salpingo-oopherectomy and pelvic lymphadenectomy	6 years	Laparoscopic reduction of internal hernia, and division of external iliac vein	Ileum herniating under the external iliac vein	Right external iliac vein	Not closed	13
Zhang et al. 2021 [19]	46-year-old female, cervical cancer	Radical Trachelectomy + Laparoscopic pelvic lymphadenectomy	9 years	Laparoscopic to open reduction of internal hernia	10 cm ischemic ileum which was viable after warming	Left external iliac artery	Suture closure	4
Chowdary et al. 2022 [20]	74-year-old male, bladder cancer	Robotic radical cystectomy and pelvic lymphadenectomy with ileal conduit formation	4 years	Open procedure, small bowel resection with anastomosis	7 cm gangrenous ileal closed loop obstruction	Right external iliac artery	Not closed	14
Allan et al. 2023 [21]	75-year-old male, bladder cancer	Radical cystectomy	4 months	Open procedure, small bowel resection with anastomosis. Primary repair of iatrogenic injury to the left external iliac artery	Ischemic ileum herniating under artery which was thought to be a band and divided.	Left external iliac artery	Peritoneal flaps	Not reported
Zanca et al. 2023 [22]	77-year-old female, endometrial cancer	Laparoscopic hysterectomy and adenexectomy with ePLND	20 months	Laparoscopic small bowel resection and anastomosis	70 cm necrotic small bowel herniated	Right external iliac artery	Absorbable mesh	8
Khalid et al. 2023 [6]	76-year-old male, bladder and prostate cancer	Robot-assisted cystoprostatectomy with ileal conduit formation and pelvic lymphadenectomy	3 years	Laparoscopic to open small bowel resection with anastomosis	40 cm herniated necrotic ileum	Right external iliac artery	Peritoneal flap and appendices epiplocae	8
Chaconas et al. 2023 [23]	74-year-old female, ovarian cancer	Hysterectomy and bilateral salpingo-oopherectomy	20 years	Open procedure, reduction of hernia. Primary repair of iatrogenic injury to left external iliac artery	Ileum volvulized underneath the artery which was thought to be a band and divided.	Right external iliac artery	Suture closure	7

Discussion

**Figure 2:**
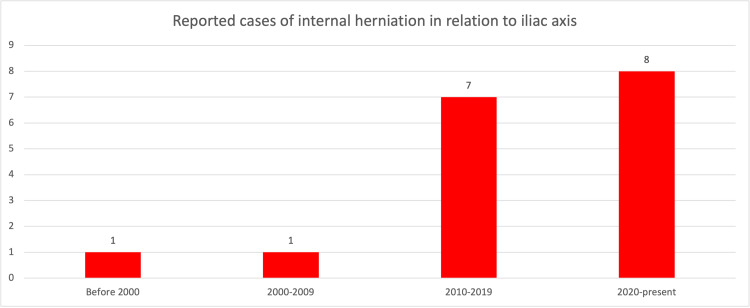
Reported cases of internal herniation of small bowel underneath iliac vasculature.

Post-operative adhesions remain the most common cause of small bowel obstruction [24]. Internal hernias are rare, and herniations related to the retroperitoneal iliac axis are even rarer. In our review, we found 17 cases of internal herniation underneath the iliac vasculature. The mean age of the patients was 63.1(±11.9) years. Our study suggests that the incidence of internal hernias has been increasing. While only two cases of such herniation have been reported before 2010, 15 have been reported since then with eight being reported in the last three years alone (Figure 2). 

In all 17 cases, the patients had a previous history of oncological resections with pelvic lymph node dissection. The latency period between initial surgery and obstruction ranged from 3 months to 20 years, with a median duration of 20 months. Seven of these previous surgeries were robotic-assisted, five were laparoscopic and four were open. 

On presentation with symptoms of small bowel obstruction, the most common approach taken was open exploration of the abdomen (n=10). Four authors started with diagnostic laparoscopy but later converted to open, while three completed the procedure laparoscopically. Small bowel resection was carried out by 13 authors with 11 reporting a primary anastomosis. Ten cases of resection-anastomosis were done by open procedure while Zanca et al. [22] completed the procedure laparoscopically. Guba et al. [8] and Frostberg [16] made an ileostomy which was later reversed. On reduction of ischemic small bowel hernia, Dumont and Wexels [10] and Zhang et al. [19] found the small bowel to be viable after warming it, so a resection was not done. Similarly, Hishikawa et al. [18] and Chaconas et al. [23] did not have to resect the small bowel as it was not ischemic. The mean hospital length of stay was 12.7 days for the 13 articles that reported it. 

**Figure 3:**
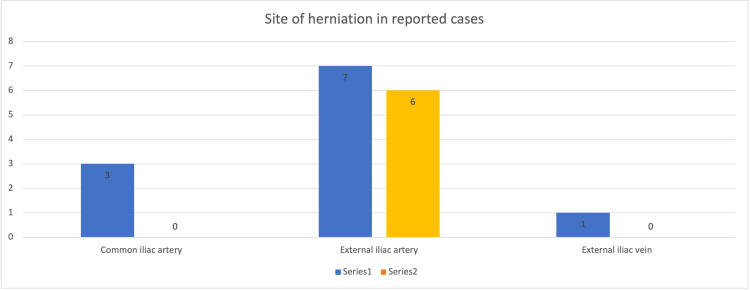
Site of herniation in cases reported.

In our study, we found that 11 internal hernias were on the right side while six were found on the left (Figure 3). All six left-sided, and seven right-sided internal hernias were underneath the external iliac artery. Three cases of herniation were reported underneath the common iliac while one case was reported underneath the external iliac vein.

In our study, we found five instances of vascular complications necessitating intervention. Guba et al. [8] found an atheromatous plaque on the right iliac artery which thrombosed 24 h postoperatively leading to acute limb ischemia and necessitating a femoro-femoral bypass surgery. Intraoperatively, Viktorin-Baier et al. [13] suspected the external iliac artery to be thrombosed due to absent distal pulsations. They proceeded to do an arterial resection with end-to-end anastomosis. A similar resection-anastomosis was performed by two other authors when they divided the external iliac artery after mistaking it for a band [21, 23]. 

In the absence of guidelines or a well-known technique to address this kind of hernia defect after the reduction of the small bowel, multiple approaches have been documented (Table 2). The most common approach was to not close the defect. Although controversial due to the chances of recurrence, the defect was not closed by three authors due to fear of vascular injury [10, 15-16]. Chowdary and Wright [20] left the defect open due to concerns regarding bacterial contamination of the closing material. Hishikawa et al. [18] chose not to close the defect because it divided the external iliac vein itself, underneath which the bowel was herniating. The second most common approach was to close the hernia defect with peritoneal flaps [6, 12, 21] and suture close [17, 19, 23]. Absorbable suture material was reported in two of the reports. A free peritoneal graft was used by Guba et al. [8] and Kim et al. [9], while a collagen patch was used by Viktorin-Baier et al. [13] and Ardelt et al. [11]. Zanca et al. [22] used an absorbable mesh to close the defect. 

**Table 2:** Intraoperative approach taken toward hernia defects.

Method of closure	Frequency
Not closed	5
Peritoneal flaps	3
Suture closure	3
Free peritoneal graft	2
Collagen patch	2
Absorbable mesh	1
Not reported	1

Most authors attributed the internal herniation of small bowel to the absence of peritoneum over the iliac vasculature, this was due to prior pelvic lymph node dissection. Extended pelvic lymph node dissection is carried out in cases of pelvic malignancies for staging, prognostic, and therapeutic purposes. This involves dissection around the iliac vasculature. Several known complications of extended pelvic lymph node dissection (ePLND) have been described in the literature, including pelvic lymphocele, postoperative ileus, neuropraxia, deep vein thrombosis, and pulmonary embolism [25]. However, internal herniation is not a well-known complication of PLND.

The authors’ personal experience with internal herniation of the small bowel underneath the external iliac artery was reported in the case report from Khalid et al. [6]. In this report, it was highlighted that intraoperatively, the artery could appear similar to a band which could lead to the iatrogenic division of a major vessel. Unfortunately, both Allan et al. [21] and Chaconas et al. [23] did report iatrogenic injury to external iliac arteries. Both these vessels appeared as a band and were divided which necessitated primary anastomosis of the vessel with vascular surgery input. This highlights the need for awareness among surgical units regarding this complication that is being reported in our study. Internal herniation underneath the iliac vasculature should be suspected in patients who have a history of pelvic lymph node dissection and have presented with small bowel obstruction.

The increasing number of patients presenting with this rare complication warrants a revisit to the operative techniques employed in pelvic surgery. Closure of the peritoneum to obliterate any potential defect should be considered during pelvic surgery which involves dissection around the iliac axis. It is worth noting that the cases highlighted in this study are those patients who presented with a strangulated or incarcerated hernia, and it is likely that there is a population of patients with similar hernias who are asymptomatic.

Although paraduodenal hernias are classically considered to be the most prevalent type of internal hernias [2]. However, more recent literature shows the increasing incidence of paramesenteric hernias; this is likely due to increasing bariatric surgeries [26]. This corresponds to the authors’ experience, and more cross-sectional studies are needed to identify the true prevalence of different types of internal hernias. 

## Conclusions

Internal hernias behind iliac arteries are rare, but their incidence is increasing. This diagnosis should be considered in patients presenting with symptoms of bowel obstruction following pelvic surgery, especially where there are tortuous iliac vessels. Be wary of intraoperative misidentification and casual division of the causative “band”! Closure of the peritoneum after pelvic lymph node dissection should be considered. More studies are needed to understand the real incidence of internal hernias after ePLND.
